# Supplementary material for: Stage-dependent differential influence of metabolic and structural networks on memory across Alzheimer’s disease continuum
Source: eLife. 2022 Sep 2;11:e77745. doi: 10.7554/eLife.77745 (PMC9477498; doi:10.7554/eLife.77745)
Supplement: Supplementary file 1. [file elife-77745-supp1.docx]

**Supplementary Table 1. Participants demographics for network seed definition step.**

|  | **Main dataset** | | |  | **Validation dataset 1** | | |  | **Validation dataset 2** | | |
| --- | --- | --- | --- | --- | --- | --- | --- | --- | --- | --- | --- |
|  | **CN** | **probable AD** | **p-value** |  | **CN** | **probable AD** | **p-value** |  | **CN** | **probable AD** | **p-value** |
| **N** | 232 | 167 | - |  | 383 | 360 | - |  | 152 | 190 | - |
| **Age, y** | 56.53~90.22 | 55.96~90.50 |  |  | 56.53~93.80 | 55.33~90.50 |  |  | 62.24~93.80 | 55.33~90.06 |  |
|  | 73.52±6.27 | 74.35±8.07 | 0.25 |  | 74.90±6.35 | 75.48±7.62 | 0.26 |  | 77.08±5.88 | 76.48±7.10 | 0.41 |
| **Gender (M/F)** | 110/122 | 99/68 | 0.02* |  | 195/188 | 214/146 | 0.02* |  | 85/65 | 115/77 | 0.55 |
| **Handedness (R/L)** | 207/25 | 154/13 | 0.32 |  | 346/37 | 336/24 | 0.14 |  | 138/12 | 181/11 | 0.41 |
| **Education, y** | 16.23±2.54 | 15.95±2.68 | 0.01* |  | 16.31±2.77 | 15.66±2.87 | 0.002* |  | 15.85±3.03 | 15.40±3.01 | 0.17 |
| **APOE e4 (+/-)** | 158/74 | 53/114 | <0.001* |  | 274/108 | 120/239 | <0.001* |  | 116/34 | 67/125 | <0.001* |
| **Memory** | 1.07±0.62 | -0.82±0.59 | <0.001* |  | 1.02±0.60 | -0.85±0.61 | <0.001* |  | 0.95±0.57 | -0.87±0.62 | <0.001* |
| **MMSE** | 29.01±1.23 | 23.30±2.37 | <0.001* |  | 29.02±1.20 | 22.83±3.36 | <0.001* |  | 29.03±1.14 | 22.37±4.13 | <0.001* |
| **CDR-SOB** | 0.05±0.16 | 4.59±1.70 | <0.001* |  | 0.02±0.47 | 4.77±2.08 | <0.001* |  | 0.14±0.71 | 4.95±2.36 | <0.001* |

Note: Data on age are range and mean ± SD. Data on education and memory are mean ± SD. Data on memory are in z-scores. Abbreviations: CN = cognitively normal; MCI = mild cognitive impairment; AD = Alzheimer's disease; A= β-amyloid; T = tau; ‘+’ = positive; ‘-’ = negative; y = years; M = male; F = female; R = right; L = left; MMSE = Mini-Mental State Exam; CDR-SOB = Clinical Dementia Rating scale-sum of box. * indicate significant group difference between CN and probable AD.
