# Supplementary material for: Stage-dependent differential influence of metabolic and structural networks on memory across Alzheimer’s disease continuum
Source: eLife. 2022 Sep 2;11:e77745. doi: 10.7554/eLife.77745 (PMC9477498; doi:10.7554/eLife.77745)
Supplement: Supplementary file 2. [file elife-77745-supp2.docx]

**Supplementary Table 2. Study participant demographics of the validation dataset 1 for the PLS-SVC model.**

|  | **A-T-** | | |  | **A-T+** | | |  | **A+T-/A+T+** | | |
| --- | --- | --- | --- | --- | --- | --- | --- | --- | --- | --- | --- |
|  | **CN** | **MCI** | **probable AD** |  | **CN** | **MCI** | **probable AD** |  | **CN** | **MCI** | **probable AD** |
| **N** | 56 | 82 | 5 |  | 92 | 79 | 11 |  | 114 | 264 | 156 |
| **Age, y** | 62.24~90.13 | 56.08~88.51 | 69.56~90.50 |  | 56.53~93.80 | 55.15~88.98 | 60.79~81.37 |  | 60.19~90.08 | 55.38~91.57 | 55.96~90.46 |
|  | 74.0±6.09^m^ | 70.62±7.53^cd^ | 79.51±9.22^m^ |  | 72.81±6.36^m^ | 70.54±8.41^cd^ | 76.22±6.52^m^ |  | 75.65±6.33^m^ | 73.70±6.79^c^ | 74.24±7.88 |
| **Gender (M/F)** | 29/27 | 45/37 | 5/0 |  | 52/40^d^ | 40/39^d^ | 10/1^cm^ |  | 53/61 | 154/110 | 89/67 |
| **Handedness (R/L)** | 53/3 | 68/14 | 5/0 |  | 81/11 | 71/8 | 10/1 |  | 107/7 | 240/24 | 144/12 |
| **Education, y** | 16.23±2.90 | 16.40±2.58 | 15.60±4.39 |  | 16.71±2.75 | 16.01±2.69 | 16.27±2.41 |  | 16.27±2.56 | 16.11±2.75 | 15.85±2.65 |
| **APOE e4 (+/-)** | 9/47 | 17/65 | 0/5 |  | 17/75 | 18/61 | 1/10 |  | 49/65^md^ | 160/104^cd^ | 113/43^cm^ |
| **Memory** | 1.06±0.66^md^ | 0.69±0.64^cd^ | -0.35±0.78^cm^ |  | 1.12±0.57^md^ | 0.53±0.62^cd^ | -0.54±0.64^cm^ |  | 0.95±0.61^md^ | 0.17±0.63^cd^ | -0.88±0.53^cm^ |
| **MMSE** | 28.93±1.44^d^ | 28.52±1.34^d^ | 25.40±2.19^cm^ |  | 28.96±1.19^md^ | 28.27±1.65^cd^ | 24.0±2.05^cm^ |  | 29.07±1.15^md^ | 27.77±1.82^cd^ | 22.89±2.67^cm^ |
| **CDR-SOB** | 0.05±0.18^md^ | 1.23±0.64^cd^ | 4.20±2.66^cm^ |  | 0.05±0.15^md^ | 1.22±0.75^cd^ | 4.32±1.23^cm^ |  | 0.04±0.15^md^ | 1.52±0.91^cd^ | 4.73±1.85^cm^ |
| **ICV** | 1564.58± 148.69 | 1528.79± 131.03 | 1583.20± 99.88 |  | 1554.05± 135.70 | 1559.29± 145.90 | 1599.97± 183.02 |  | 1536.14± 145.69 | 1567.10± 148.96.52 | 1551.78± 161.75 |

Note: Data on age are range and mean ± SD. Data on education, ICV and memory are mean ± SD. Data on memory are in z-scores. Abbreviations: CN = cognitively normal; MCI = mild cognitive impairment; AD = Alzheimer's disease; A= β-amyloid; T = tau; ‘+’ = positive; ‘-’ = negative; y = years; M = male; F = female; R = right; L = left; MMSE = Mini-Mental State Exam; CDR-SOB = Clinical Dementia Rating scale-sum of box; ICV = intracranial volume. Superscripts (‘^c^’, ‘^m^’, ‘^d^’) represent significant group difference with CN, MCI and probable AD respectively.
