# Supplementary material for: Stage-dependent differential influence of metabolic and structural networks on memory across Alzheimer’s disease continuum
Source: eLife. 2022 Sep 2;11:e77745. doi: 10.7554/eLife.77745 (PMC9477498; doi:10.7554/eLife.77745)
Supplement: Supplementary file 3. [file elife-77745-supp3.docx]

**Supplementary Table 3. Study participant demographics of the validation dataset 2 for the SVC model.**

|  | **A+T-/A+T+** | | |
| --- | --- | --- | --- |
|  | **CN** | **MCI** | **probable AD** |
| **N** | 32 | 39 | 91 |
| **Age, y** | 62.94~88.65 | 62.47~85.09 | 59.01~89.94 |
|  | 77.46±6.07 | 76.84±5.04 | 75.76±6.84 |
| **Gender (M/F)** | 18/14 | 27/12 | 54/37 |
| **Handedness (R/L)** | 32/0 | 37/2 | 85/6 |
| **Education, y** | 15.75±3.45 | 15.92±2.90 | 15.52±3.09 |
| **APOE e4 (+/-)** | 14/18 | 19/20 | 72/19 |
| **Memory** | 0.93±0.56 | -0.01±0.58 | -0.89±0.58 |
| **MMSE** | 29.25±1.05 | 27.38±1.65 | 22.01±3.93 |
| **CDR-SOB** | 0.03±0.12 | 1.59±0.99 | 4.92±2.30 |
