# Supplementary material for: Stage-dependent differential influence of metabolic and structural networks on memory across Alzheimer’s disease continuum
Source: eLife. 2022 Sep 2;11:e77745. doi: 10.7554/eLife.77745 (PMC9477498; doi:10.7554/eLife.77745)
Supplement: Supplementary file 4. [file elife-77745-supp4.docx]

**Supplementary Table 4. The coordinates of the peak foci of regions showing difference in metabolism and grey matter volume between probable AD and healthy controls.**

|  |  |  | **Main dataset** | | |  | **Validation dataset 1** | | |  | **Validation dataset 2** | | |
| --- | --- | --- | --- | --- | --- | --- | --- | --- | --- | --- | --- | --- | --- |
| **Network Label** | **Anatomical Label** |  | **x** | **y** | **z** |  | **x** | **y** | **z** |  | **x** | **y** | **z** |
| Hippocampus | left HIP |  | -19 | -3 | -22 |  | -33 | -18 | -17 |  | -18 | -36 | 1 |
|  | right HIP |  | 19 | -3 | -21 |  | 19 | -2 | -22 |  | 18 | -3 | -22 |
| Default mode | left ANG |  | -47 | -63 | 39 |  | -46 | -63 | 39 |  | -49 | -58 | 26 |
|  | right ANG |  | 50 | -60 | 37 |  | 51 | -60 | 37 |  | 54 | -46 | 38 |
|  | PCC |  | -4 | -52 | 29 |  | -4 | -52 | 27 |  | 0 | -35 | 31 |
|  | mPFC |  | 1 | 50 | -5 |  | -2 | 57 | 4 |  | -2 | 36 | 27 |
| Salience | left INS |  | -43 | 0 | -10 |  | -43 | -4 | -5 |  | -29 | 7 | -21 |
|  | right INS |  | 44 | -2 | -7 |  | 44 | -2 | -5 |  | 45 | -3 | 0 |
| Executive control | left DLPFC |  | -46 | 11 | 40 |  | -46 | 11 | 40 |  | -27 | 23 | 45 |
|  | right DLPFC |  | 42 | 15 | 40 |  | 42 | 15 | 40 |  | 29 | 37 | 41 |
|  | left PPC |  | -53 | -45 | 43 |  | -52 | -46 | 45 |  | -44 | -66 | 41 |
|  | right PPC |  | 52 | -52 | 44 |  | 52 | -52 | 44 |  | 52 | -60 | 37 |

Abbreviations: HIP = hippocampus; ANG = angular gyrus; PPC = posterior parietal cortex; mPFC = medial prefrontal cortex; INS = insular; DLPFC = dorsolateral prefrontal cortex; PCC = posterior cingulate cortex.
