# Supplementary material for: Stage-dependent differential influence of metabolic and structural networks on memory across Alzheimer’s disease continuum
Source: eLife. 2022 Sep 2;11:e77745. doi: 10.7554/eLife.77745 (PMC9477498; doi:10.7554/eLife.77745)
Supplement: Transparent reporting form [file elife-77745-transrepform1.docx]

***eLife’s* transparent reporting form**

We encourage authors to provide detailed information *within their submission* to facilitate the interpretation and replication of experiments. Authors can upload supporting documentation to indicate the use of appropriate reporting guidelines for health-related research (see [EQUATOR](http://www.equator-network.org/) [Network](http://www.equator-network.org/)), life science research (see the [BioSharing Information Resource](https://biosharing.org/)), or the ARRIVE guidelines for reporting work involving animal research. Where applicable, authors should refer to any relevant reporting standards documents in this form.

If you have any questions, please consult our Journal Policies and/or contact us: [editorial@elifesciences.org.](mailto:editorial@elifesciences.org)

# Sample-size estimation

- You should state whether an appropriate sample size was computed when the study was being designed
- You should state the statistical method of sample size computation and any required assumptions
- If no explicit power analysis was used, you should describe how you decided what sample (replicate) size (number) to use

Please outline where this information can be found within the submission (e.g., sections or figure

legends), or explain why this information doesn’t apply to your submission:

**Information about our sample size derivation can be found in P.29, Methods - participants.**

**We selected as many participants as possible from the publicly available ADNI database, one of the most extensive database on dementia with information on Tau and Amyloid, based on their demographics (in accord to ADNI criteria) and MRI data quality.**

**Specifically, we included 708 participants (195 CN, 374 MCI and 139 probable AD) in the main study cohort, and 859 participants (262 CN, 425 MCI and 172 probable AD) in the validation dataset. These participants were projected into the partial least square analysis to derive the brain structural/metabolic covariance networks. Based on our previous work on network derivation analyses using partial least square (Veldsman M, 2020), a sample size of more than 100 is adequate. In our sparse varying coefficient models, ordered participants were distributed evenly into bins, with 10 subjects in each bin. Previous study (Daye ZJ, 2012) has proved 10 samples per bin is adequate to make reliable estimation.**

**Veldsman M, Cheng HJ, Ji F, Werden E, Khlif MS, Ng KK, Lim JK, Qian X, Yu H, Zhou JH, Brodtmann A. Degeneration of structural brain networks is associated with cognitive decline after ischaemic stroke. Brain communications. 2020;2(2).**

**Daye ZJ, Xie J, Li H. A sparse structured shrinkage estimator for nonparametric varying-coefficient model with an application in genomics. Journal of Computational and Graphical Statistics. 2012 Jan 1;21(1):110-33.**

# Replicates

- You should report how often each experiment was performed
- You should include a definition of biological versus technical replication
- The data obtained should be provided and sufficient information should be provided to indicate the number of independent biological and/or technical replicates
- If you encountered any outliers, you should describe how these were handled
- Criteria for exclusion/inclusion of data should be clearly stated
- High-throughput sequence data should be uploaded before submission, with a private link for reviewers provided (these are available from both GEO and ArrayExpress)

Please outline where this information can be found within the submission (e.g., sections or figure

legends), or explain why this information doesn’t apply to your submission:

**The information could be found in the sections “Results: Replication in the validation dataset” and “Methods: Participants”, and Supplementary Figure 1. To replicate the findings, we repeated the same analyses using another larger validation dataset by adding additional 468 individuals and observed similar results. Moreover, our findings were robust when we repeated the analyses with another ordering strategy of merging both MCI and dementia stages.**

**Statistical reporting**

- Statistical analysis methods should be described and justified
- Raw data should be presented in figures whenever informative to do so (typically when N per group is less than 10)
- For each experiment, you should identify the statistical tests used, exact values of N, definitions of center, methods of multiple test correction, and dispersion and precision measures (e.g., mean, median, SD, SEM, confidence intervals; and, for the major substantive results, a measure of effect size (e.g., Pearson's r, Cohen's d)
- Report exact p-values wherever possible alongside the summary statistics and 95% confidence intervals. These should be reported for all key questions and not only when the p-value is less than 0.05.

Please outline where this information can be found within the submission (e.g., sections or figure

legends), or explain why this information doesn’t apply to your submission:

**Details about our statistical analyses can be found in P.31-35, Statistical Analyses; A schematic and flow chart about the analysis and sample sizes used in each of our analyses can be found in P.10 Figure 1 and P.53 Supplementary Figure 1. A schematic of how data were inputted into our SVC model can be found in P.58 Supplementary Figure 4.**

(For large datasets, or papers with a very large number of statistical tests, you may upload a single table file with tests, Ns, etc., with reference to sections in the manuscript.)

# Group allocation

- Indicate how samples were allocated into experimental groups (in the case of clinical studies, please specify allocation to treatment method); if randomization was used, please also state if restricted randomization was applied
- Indicate if masking was used during group allocation, data collection and/or data analysis

Please outline where this information can be found within the submission (e.g., sections or figure legends), or explain why this information doesn’t apply to your submission:

**Our control and patient group definitions and cutoffs for Amyloid and Tau pathologies can be found in P.29-30 Participants; the demographical details of these groups can be found in P.12, Table 1 and P.49-50, Supplementary Table 1 and 2. Summary of the control and patient groups used in the analyses can be found in P.12 Table 1 and P.49-50, Supplementary Table 1 and 2.**

# Additional data files (“source data”)

- We encourage you to upload relevant additional data files, such as numerical data that are represented as a graph in a figure, or as a summary table
- Where provided, these should be in the most useful format, and they can be uploaded as

“Source data” files linked to a main figure or table

- Include model definition files including the full list of parameters used
- Include code used for data analysis (e.g., R, MatLab)
- Avoid stating that data files are “available upon request”

Please indicate the figures or tables for which source data files have been provided:

**Summary of the control and patient groups used in the analyses can be found in P.12 Table 1 and P.49-50, Supplementary Table 1 and 2f**
